# Immune imbalance in rheumatoid arthritis: insights from γδ T cell receptor phenotyping

**DOI:** 10.1007/s13577-026-01393-1

**Published:** 2026-06-04

**Authors:** Sylwia Biały, Joanna Wielińska, Kinga Maria Tyczyńska, Jerzy Świerkot, Katarzyna Bogunia-Kubik

**Affiliations:** 1 Laboratory of Clinical Immunogenetics and Pharmacogenetics, Hirszfeld Institute of Immunology and Experimental Therapy, Polish Academy of Sciences, Wroclaw, Poland; 2 Department of General Biochemistry, University of Lodz, Lodz, Poland; 3 Department and Clinic of Rheumatology and Internal Diseases, Wroclaw Medical University, Wroclaw, Poland

**Keywords:** Rheumatoid arthritis, γδ T cells, TNF inhibitors, Surface receptors, Immunophenotype

## Abstract

Rheumatoid arthritis (RA) is a chronic autoimmune disease characterized by persistent synovial inflammation and progressive joint destruction. While conventional αβ T cells have been extensively studied, the contribution of γδ T cells to RA pathogenesis remains insufficiently understood. Unlike αβ T cells, γδ T cells recognize antigens independently of major histocompatibility complex (MHC) restriction, and their surface receptor expression plays a pivotal role in regulating effector functions. This study aimed to investigate the phenotype and surface receptor profile of γδ T cells in RA patients undergoing anti-TNF therapy, to better understand their potential immunoregulatory and prognostic significance. Peripheral blood samples were collected from RA patients before and during treatment with TNF inhibitors, as well as from healthy controls. Flow cytometry was used to quantify γδ T cell frequency and to assess the expression of selected activation, differentiation, and exhaustion markers on their surface. RA patients exhibited a significant reduction in the frequency of circulating γδ T cells compared with healthy controls. Moreover, γδ T cells in RA displayed phenotypic features of advanced differentiation and functional exhaustion, including altered expression of key surface receptors. The observed alterations in γδ T cell phenotype and receptor expression suggest chronic immune activation in RA. These findings highlight the potential of γδ T cell surface markers as candidate biomarkers for disease activity and response to anti-TNF therapy.

**Supplementary Information:**

The online version contains supplementary material available at 10.1007/s13577-026-01393-1.

## Introduction

Rheumatoid arthritis (RA) is a chronic autoimmune disease with a complex and multifactorial pathogenesis. In addition to genetic predispositions, a central element in RA pathophysiology is the disruption of the delicate balance between pro-inflammatory and anti-inflammatory cellular and molecular mechanisms. A shift toward a predominantly pro-inflammatory state triggers a cascade of events leading to acute inflammation within the joints. As the disease progresses, this persistent inflammation causes degradation of articular cartilage and bone structures, ultimately resulting in disability for individuals affected by RA [1].

Epidemiological data indicate that RA affects up to 0.2% of the global population, with a marked predominance in women, particularly in the fifth decades of life. Increasingly classified as a civilization-associated disease, RA underscores the urgent need to elucidate its underlying pathogenic mechanisms [2]. Within this context, gamma delta (γδ) T cells represent an intriguing focus of investigation. These unconventional T lymphocytes exhibit functional characteristics of both innate and adaptive immune cells, acting as a bridge between the two arms of immunity and thus playing a unique role in maintaining immunological homeostasis [3, 4]. Notably, γδ T cells do not require antigen presentation through the major histocompatibility complex (MHC) for activation, highlighting the importance of their broad repertoire of surface receptors. Many of these receptors are shared with natural killer (NK) cells, including members of the NCR, NKG2, and KIR families, enabling γδ T cells to perform NK-like functions [5]. For example, similar to NK cells, γδ T lymphocytes are capable of both direct and indirect cytotoxicity [6].

The role of γδ T cells in RA remains incompletely understood; however, several studies suggest their potential involvement in disease pathogenesis. Altered levels of γδ T cells have been observed in both the peripheral blood and synovium of RA patients compared to healthy controls [7]. Moreover, increased expression of HLA-DR on circulating γδ + T cells has been correlated with rheumatoid arthritis disease activity [8], while β-chain amino acid sequence of HLA-DR—known as the shared epitope (SE)—is associated with increased susceptibility to RA [9, 10]. Given their capacity to modulate inflammatory processes, γδ T cells could represent a valuable parameter for monitoring disease activity and therapeutic response, particularly in the context of biologic treatments.

Further complexity arises from the existence of distinct γδ T cell subpopulations, defined by their T-cell receptor δ-chain usage [11]. The most common subsets in adults are Vδ1 cells, predominantly found in tissues, and Vδ2 cells, primarily circulating in the peripheral blood. These subsets differ in terms of function and activity in RA. Notably, Vδ2 cells have been reported to migrate from the bloodstream to the synovium, where they may contribute to ongoing inflammation [12]. However, their precise role in this context remains to be fully elucidated.

In recent years, the concept of the RA disease continuum has gained particular significance in studies of pathogenesis and diagnosis. It suggests that the disease process begins long before the appearance of clinical symptoms, and that autoantibodies such as rheumatoid factor (RF) and anti-citrullinated protein antibodies (ACPA) can already be detected in the early phase of disease [13]. The presence of these autoantibodies may precede the onset of full-blown RA by several years, making them highly useful for identifying individuals at high risk [14]. Despite their strong predictive value, not all seropositive individuals go on to develop the disease, highlighting the need for additional biomarkers to support diagnosis and patient stratification, as demonstrated in recent studies [15]. Cellular biomarkers, including surface receptors on immune cells, may represent promising tools for assessing the degree of immune activation, which could have direct implications for monitoring response to biologic therapies [15].

In this context, in our previous studies we have repeatedly highlighted the significant role of genetic variants, particularly single nucleotide polymorphisms (SNPs), which may be associated with disease susceptibility, disease activity, as well as response to biologic therapy. Among the analyzed markers, we included variability in genes encoding cytokines, surface receptors, classical and non-classical HLA molecules, as well as miRNA molecules [10, 16–20]. Importantly, some of these molecules are also expressed on γδ T cells, suggesting their potential involvement in mechanisms regulating the inflammatory response and the effectiveness of biologic therapy [21]. However, the role of γδ T cells in the pathogenesis of RA and in shaping the response to biologic treatment remains insufficiently understood.

Therefore, the aim of this study was to evaluate the functional potential of γδ T cells by analyzing the expression of selected surface receptors on these cells in patients with RA undergoing biologic therapy, and to assess its association with therapeutic response.

## Materials and methods

### Study population

The study enrolled 34 patients diagnosed with RA according to the 2010 European League Against Rheumatism (EULAR) classification criteria. All patients received the tumor necrosis factor (TNF) inhibitor under the Polish National Health Fund (NFZ) drug program B.33 *Treatment of Rheumatoid Arthritis and Juvenile Idiopathic Arthritis with an Aggressive Course (ICD-10 M05, M06, M08)*, thereby meeting the inclusion criteria (described below). For some patients, this represented a subsequent line of biological therapy following a prior biologic agent; a mandatory minimum drug washout period of two months was required before inclusion. Patients were evaluated at three defined time points: (I) at therapy qualification (baseline) (T = 0), (II) after three months (T = 3), and (III) six months (T = 6) of anti-TNF treatment. Assessment of treatment response in patients with RA is based on the analysis of the change in disease activity score DAS28 (ΔDAS28) and the current level of disease activity after initiation of therapy. A good response is defined as a decrease in DAS28 of at least 1.2 accompanied by the achievement of low disease activity, indicating effective treatment. A moderate response is observed when the DAS28 decreases by more than 0.6 but less than 1.2 with low or moderate disease activity, or when the decrease is at least 1.2 but disease activity remains moderate or high. No response to treatment is defined as a decrease in DAS28 of less than 0.6, or when the improvement is insufficient (less than 1.2) and high disease activity persists, indicating the need for modification of therapy. A detailed demographic and clinical characterization of the study group is presented in Table 1.

**Table 1:** Characteristics of RA patients undergoing biological therapy enrolled in the study.

Characteristics	RA patients	Healthy controls
Number (n)	34	44
Female (%)	67.65%	73.33%
Age [years], mean ± SD	57.97 ± 12.85	44.43 ± 9.25
Disease onset [years], mean ± SD	47.00 ± 14.20	n/a
BMI [kg/m^2^], mean ± SD	25.85 ± 3.84	n/a
RF positive (%)	63.64%	n/a
Anti-CCP positive (%)	56.25%	n/a
DAS28 before treatment, mean ± SD	5.86 ± 0.81	n/a
Anti-TNF Treatment [% of patients]		
Etanercept (%)	32.35%	n/a
Adalimumab (%)	41.18%	n/a
Certolizumab pegol (%)	17.65%	n/a
Golimumab (%)	8.82%	n/a

Inclusion criteria were as follows: age ≥ 18 years, absence of severe comorbid conditions or any history of malignancy, no current substance abuse, not pregnant or breastfeeding at the time of enrollment. All participants provided written informed consent for participation in the study, which was approved by the Bioethics Committee at the Hirszfeld Institute of Immunology and Experimental Therapy, Polish Academy of Sciences, Wrocław, Poland (Approval No. KB-2/2024). All procedures involving human participants were performed in accordance with the ethical standards of the institutional and/or national research committee and with the Helsinki declaration and its later amendments or comparable ethical standards.

Furthermore, participants consented to the use of their anonymized clinical data, including the following parameters: age, sex, weight, body mass index (BMI), disease duration, Disease Activity Score (DAS28), Visual Analog Scale (VAS), Number of Swollen Joints (NSJ), Number of Tender Joints (NTJ), C-reactive protein (CRP) levels, erythrocyte sedimentation rate (ESR), hemoglobin levels, and liver function markers (creatinine, aspartate aminotransferase [AspAT], alanine aminotransferase [AlAT]).

### Healthy control group

Peripheral blood samples were also collected from 44 healthy volunteers who served as the control group (HC). Control individuals met the following criteria: age ≥ 18 years, no autoimmune diseases, and no immunomodulatory treatment. The mean age of the control group was slightly lower than that of the study group. However, this difference was not statistically significant and there was no correlation between the analyzed parameters and age. Nevertheless, it should be noted that previous studies have demonstrated age-related changes in immune cell populations; therefore, despite the lack of correlation observed in the present analysis, age-related effects cannot be entirely excluded and should be considered a limitation of this study.

### Sample collection and flow cytometry analysis

Peripheral venous blood was drawn into BD Vacutainer EDTA blood collection tubes. Fresh whole blood and of BD Horizon Brilliant Stain Buffer Plus (BD Biosciences) was used for flow cytometric staining in each patient and control subject. Initially, samples were preincubated with anti-human TCRγδ -PE-Cy7 (clone: 11F2) antibody in the dark. Next, they were stained at room temperature with combinations of the following antibodies: Vδ1-PE (clone: REA173), Vδ2-BV421 (clone: B6), CD4-APC (clone: SK3), CD3-APC-H7 (clone: SK7), CD8-BV510 (clone: SK1), CD28-BB515 (clone: CD28.2), CD16-PE (clone: B73.1), CD56-BB700 (clone: NCAM16.2), CD27-APC (clone: M-T271), PD-1(CD279)-BV429 (clone: EH12.1), CD45RA-BV480 (clone: HI100), TIM-3(CD366)-BB515 (clone: 7D3), NKp30(CD337)-PE (clone: P30-15), TIGIT-BV421 (clone: 741,182), CD226-BV510 (clone: DX11). All antibodies used, except for Vδ1 (Miltenyi Biotec), were obtained from the manufacturer BD Biosciences.

Following monoclonal antibody staining, erythrocyte lysis was performed using 1X BD Pharm Lyse™ (BD Biosciences) with a dark incubation. Next, samples were centrifuged at 400 × g for 5 min and washed twice with phosphate-buffered saline (PBS). Finally, cells were resuspended in 250 µL PBS prior to flow cytometry analysis.

For assessment of cell viability, the 7-AAD viability dye (BD Biosciences) was added to the test tube.

### Flow cytometry data acquisition and statistical analysis

**Fig. 1:**
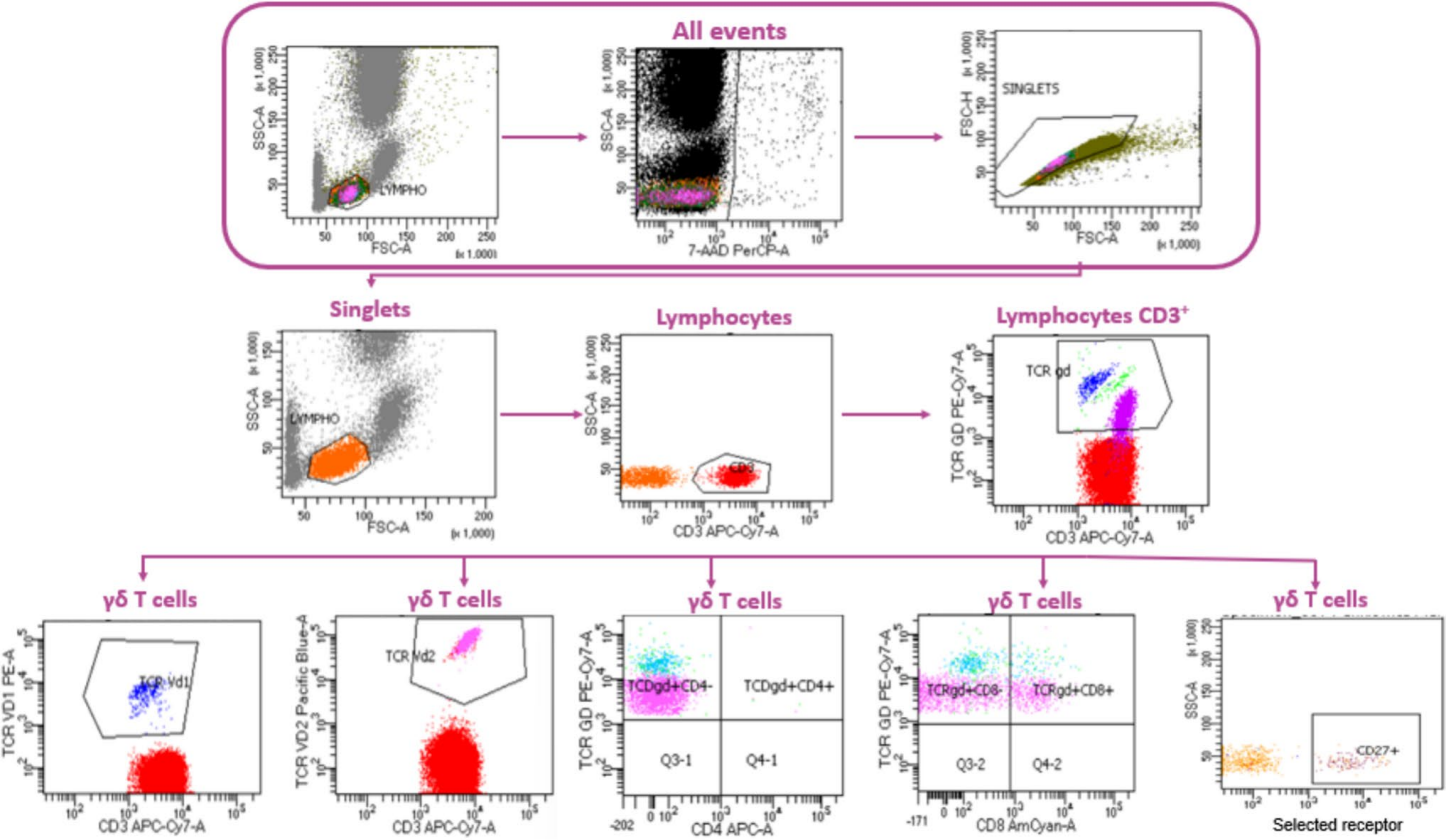
Gating strategy for cell subpopulations in the flow cytometry analysis. γδ T cells were identified from live single lymphocytes based on the expression of the γδ TCR receptor.

Data acquisition was performed using FACS Canto II equipped with DIVA software (Becton Dickinson Biosciences, Franklin Lakes, NJ, USA). Approximately 50,000 events were collected in each test tube. The BD CS&T beads (BD Biosciences) were used daily to provide quality control of the flow cytometer. Compensation was set based on the BD™ CompBeads Set Anti-Mouse Ig (BD Biosciences) and single-stained samples. The fluorescence-minus-one (FMO) controls were used for gating determination. The gating strategy for the identification and analysis of γδ T cell subsets is presented in Fig. 1.

The results of the flow cytometric analysis were compared with the clinical parameters of RA patients and subsequently subjected to detailed statistical evaluation. The proportion of γδ T lymphocytes among CD3⁺ T cells was determined according to the established gating strategy. Differences in the percentage of cells, median fluorescence intensity (MFI) values, and co-expression of the analyzed receptors between RA patients at different time points were assessed using analysis of variance (ANOVA) and its non-parametric equivalent, the Kruskal–Wallis test for non-normally distributed data. Comparisons between RA patients and healthy controls were performed using the Mann–Whitney U test and the non-parametric equivalent of the t-test with Welch’s correction for non-normally distributed data.

Correlation matrices were then constructed to assess the relationships between the proportion of γδ T lymphocytes expressing specific receptors and the clinical parameters of RA patients, as well as between the expression levels of different receptors.

To evaluate the ability of the analyzed γδ T cell surface receptors to discriminate RA patients from healthy individuals, receiver operating characteristic (ROC) curve analysis was performed. For each analyzed marker, the area under the curve (AUC) was calculated along with the 95% confidence interval, and the optimal cut-off value maximizing sensitivity and specificity was determined using Youden’s method. All statistical analyses were carried out using GraphPad Prism software, version 8.0.1. A p ≤ 0.05 was considered to be statistically significant.

## Results

### Characteristics of γδ T cells in RA patients compared to healthy controls

Γδ T cells were identified within the CD3⁺ lymphocyte population according to the gating strategy illustrated in Fig. 1. Among patients with RA, an average of 35.96% of γδ T cells expressed the CD8 marker. This proportion remained consistent throughout the treatment period and did not differ significantly from that observed in the healthy control group.

**Fig. 2:**
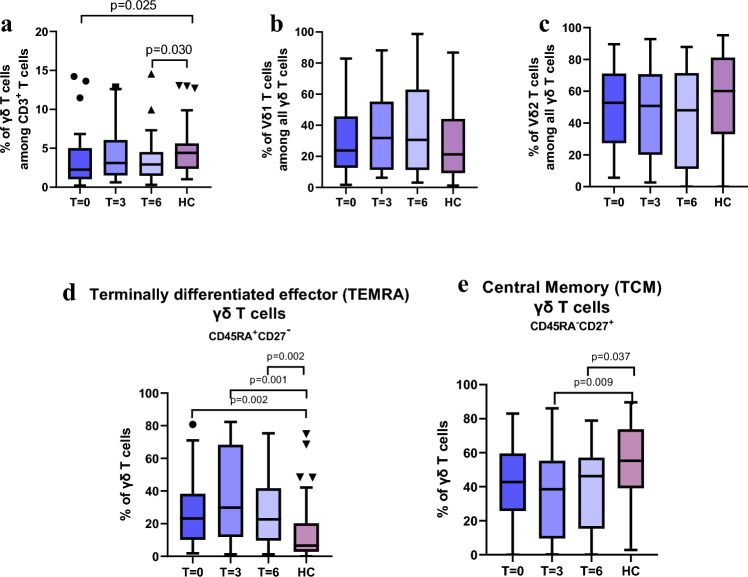
Characterization of the proportions of γδ T cells and their subpopulations in patients with rheumatoid arthritis (RA) during anti-TNF therapy at different time points: before treatment (T = 0), after 3 months of treatment (T = 3), and after 6 months of treatment (T = 6), as well as in healthy controls (HC). **a**γδ T cells among all CD3 + lymphocytes, and **b**,**c** Vδ1 and Vδ2 subpopulations among γδ T; **d** percentage of terminally differentiated effector γδ T cells (TEMRA), and **e** a percentage of central memory γδ T cells (TCM) than healthy individuals (HC)

In patients with RA, the proportion of γδ T cells was found to be significantly lower compared to healthy control subjects (Fig. 2a). This difference reached statistical significance both before the initiation of anti‑TNF therapy (p = 0.025) and after six months of treatment (p = 0.030). No such association was observed when analyzing the MFI of γδ T cells. However, differences were also observed at the level of the absolute number of γδ T cells collected among 50,000 total events. Healthy controls exhibited on average 69% more γδ T cells than patients before treatment (p < 0.001). This number increased by 55% after 3 months of treatment (p = 0.008) and by 106% after 6 months of anti-TNF therapy (p = 0.017) (data not shown).

No statistically significant differences were detected between RA patients and healthy controls when examining the proportions of Vδ1 and Vδ2 subsets, as well as their respective MFI values (Fig. 2b,c). As expected, the frequency of Vδ2 cells in peripheral blood was higher than that of Vδ1 cells.

Based on the expression of CD45RA and CD27 on the surface of γδ T cells, distinct differences in their functional phenotype were observed (Fig. 2d,e). In patients with RA, a significantly higher proportion of γδ T cells exhibiting the CD45RA⁺CD27⁻ terminally differentiated effector (TEMRA) phenotype, indicative of advanced maturation, cytotoxic potential and cellular exhaustion, was detected both before treatment (p = 0.002) and during anti‑TNF therapy (p = 0.001 and p = 0.002) compared to healthy controls (Fig. 2d). Conversely, RA patients demonstrated a reduced frequency of γδ T cells with the CD45RA⁻CD27⁺ central memory (TCM) phenotype, which are poised for rapid expansion upon re‑encounter with antigen. This difference was statistically significant during treatment (p = 0.009 and p = 0.037) compared to the control group (Fig. 2e). However, no significant differences were found in the proportions of naïve γδ T cells (CD45RA⁺CD27⁺) or effector memory (TEM) γδ T cells (CD45RA⁻CD27⁻) between RA patients and healthy individuals (see Supplementary Data Fig. S1a,b).

### Reduction in γδ T‑cell frequency and TCRγδ expression may be associated with poorer response to anti‑TNF therapy

**Fig. 3:**
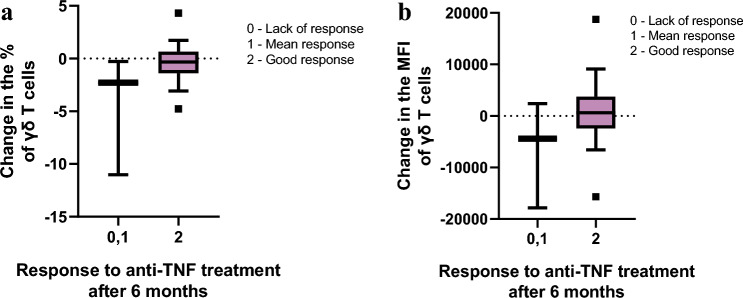
A trend indicating a relationship between changes in a) the percentage and b) the MFI of γδ T cells and the response to anti-TNF treatment after 6 months. A decrease in both the percentage and MFI of γδ T cells may be associated with a poorer response to anti-TNF therapy, as determined by changes in DAS28

Examining the relationship between anti‑TNF treatment response and changes in both the proportion of γδ T cells and the TCRγδ receptor expression levels (based on median MFI values) revealed that patients with a poorer therapeutic response after six months exhibited a decline in both γδ T‑cell frequency and MFI values compared to those who responded well to treatment (Fig. 3). While these trends did not reach statistical significance, they are noteworthy and may warrant further investigation.

### Differences in surface receptor expression on γδ T cells

Significant differences were observed in both the proportion of γδ T cells that expressed specific surface receptors (%), as well as in the MFI of these receptors. The analyzed receptors were grouped as follows: Activating receptors: CD28, CD27, NCR3 (CD337), DNAM‑1 (CD226); Inhibitory receptors: PD‑1 (CD279), TIM‑3 (CD366), TIGIT; Cytotoxicity and maturation markers: CD16, CD56, CD45RA.

#### Lower levels of activating receptors in RA patients

It was found that γδ T cells from RA patients display activating receptors on a smaller proportion (%) of cells and with lower expression intensity (MFI) compared to healthy controls. The most notable and statistically significant differences were observed for CD28 and DNAM‑1.

**Fig. 4:**
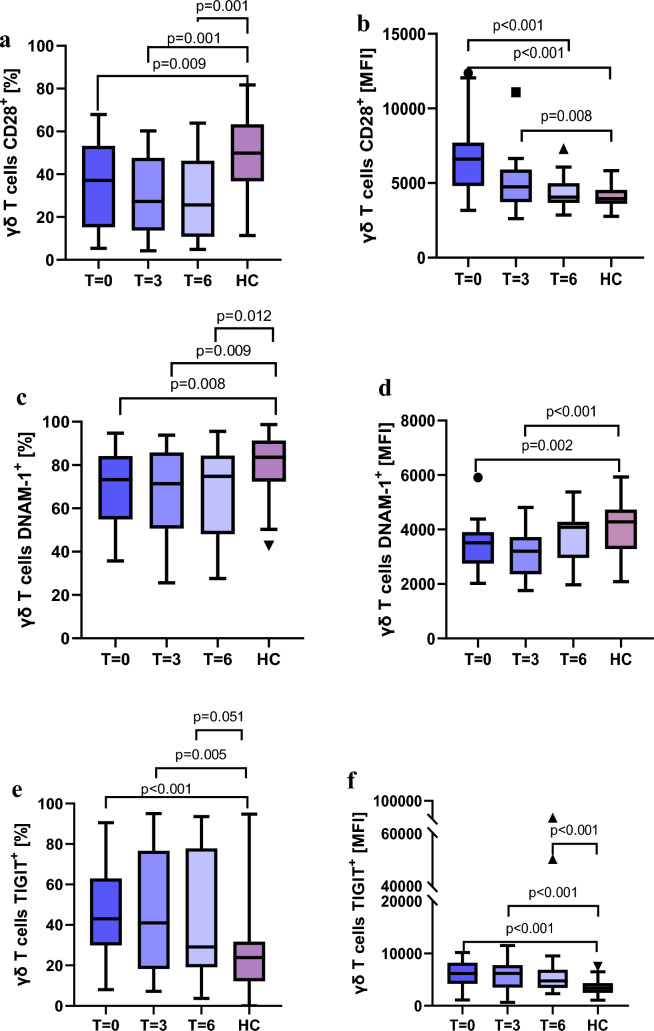
Changes in **a,c,e** the percentage and **b,d,f** the MFI of activating receptors **a,b** CD28 and **c,d** DNAM-1 s and inhibitory receptor **e,f** TIGIT during anti-TNF treatment compared to healthy control

RA patients exhibited a reduced proportion of γδ T cells expressing the CD28 receptor (p = 0.009, p = 0.001, p = 0.001) (Fig. 4a). Interestingly, CD28 MFI values were significantly higher at baseline in RA patients than in healthy controls (p < 0.001), but declined during anti‑TNF therapy (Fig. 4b).

The co‑stimulatory activating receptor DNAM‑1 was present on a significantly smaller proportion of γδ T cells in RA patients (p = 0.008, p = 0.009, p = 0.012) (Fig. 4c), with markedly lower expression intensity, compared with healthy individuals (p = 0.002, p < 0.001) (Fig. 4d).

Significant results were also obtained regarding the proportion of γδ T cells for the NCR3 receptor before treatment (p = 0.002), as well as for CD27 at all stages of therapy (p = 0.058, p = 0.015, p = 0.038) (see Supplementary Data Fig. S2a,b).

#### Higher levels of inhibitory receptors in RA patients

The most notable finding among the inhibitory receptors concerned TIGIT, which exhibited markedly elevated values in RA patients compared to healthy controls, both in terms of the proportion of γδ T cells expressing the receptor (p < 0.001, p = 0.005, p = 0.051) (Fig. 4e) and expression intensity (MFI) (p < 0.001, p = 0.001) (Fig. 4f).

Additional significant results were observed for PD‑1 MFI at all stages of treatment (p = 0.008, p = 0.038, p = 0.042). A similar trend was noted for the percentage of γδ T cells expressing TIM‑3 (see Supplementary Data Fig. S2c,d).

#### Higher levels of cytotoxicity‑ and naïve‑associated receptors

**Fig. 5:**
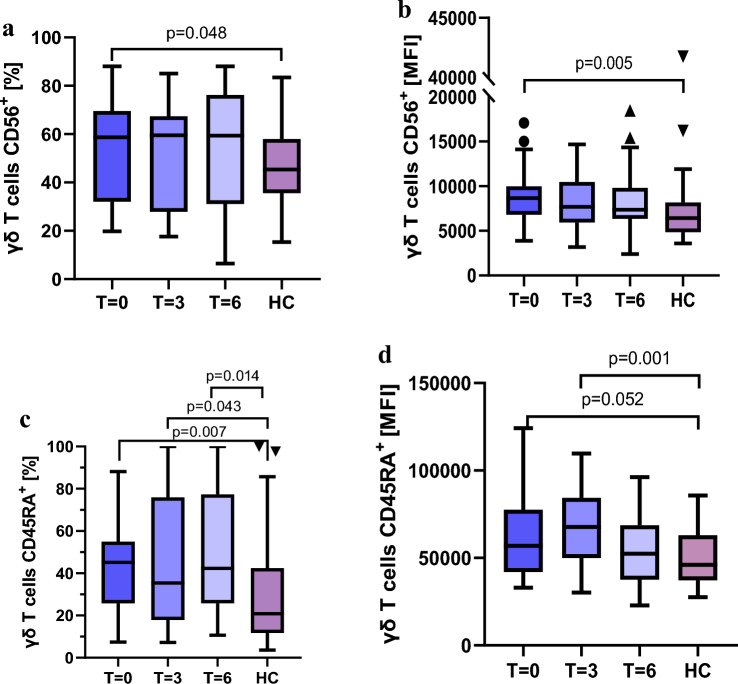
Higher values of **a,c** the percentage of γδ T cells and **b,d** the MFI of selected γδ T-cell receptors in RA patients during anti-TNF treatment compared to healthy controls; **a,b** values for the cytotoxicity receptor CD56; **c,d** values for the cell maturity marker CD45RA

The CD56 receptor, which is associated with the cytotoxic potential of γδ T cells, was found to be expressed on a significantly greater proportion of γδ T cells in RA patients before treatment (p = 0.048) (Fig. 5a). Higher expression levels were also observed, as reflected by MFI (p = 0.005), compared to healthy controls (Fig. 5b).

Similarly, a greater proportion of γδ T cells expressed CD16, another receptor involved in γδ T‑cell‑mediated cytotoxicity, in RA patients before treatment than healthy individuals (p = 0.011) (see Supplementary Data Fig. S2e).

CD45RA, a marker that reflects the maturation status of γδ T cells, also exhibited higher values for both percentage and MFI during treatment compared to healthy controls Fig. 5c,d).

### Opposing correlations between activating and inhibitory receptors and cytotoxicity‑associated markers on γδ T cells in RA patients before treatment

**Fig. 6:**
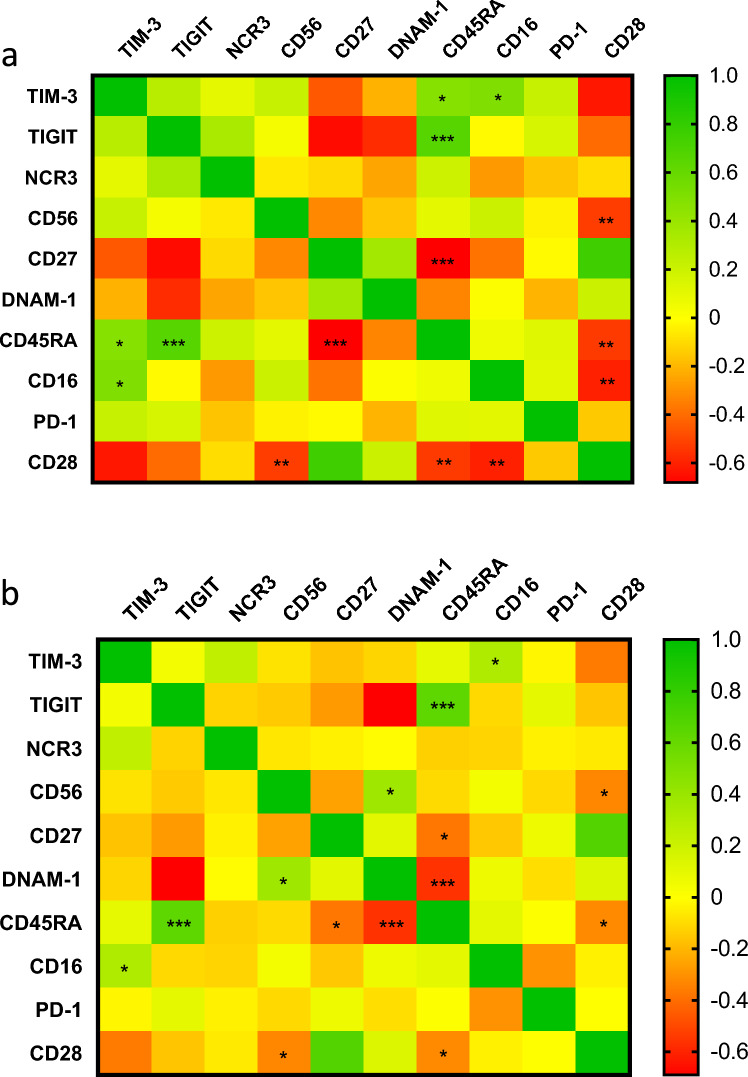
**a** Correlation matrix of the percentage of γδ T cells expressing specific surface receptors in RA patients before anti-TNF biologic treatment based on R-values. Statistical significance of the correlations was determined as *p < 0.05, **p < 0.01, ***p < 0.001. **b** Correlation matrix of the percentage of γδ T cells expressing specific surface receptors in healthy controls based on R-values. Statistical significance of the correlations was determined as *p < 0.05, **p < 0.01, ***p < 0.001

A correlation analysis was performed to investigate relationships between individual receptors expressed on the surface of γδ T cells (Fig. 6). Examining the percentage of γδ T cells expressing a given receptor revealed several notable findings, as well as the expected patterns. These included positive correlations within the activating and inhibitory receptor groups and negative correlations between them.

Specifically, negative correlations were observed between activating receptors (CD28, CD27) and receptors associated with γδ T‑cell cytotoxicity (CD56, CD16, and CD45RA). In contrast, positive correlations were found between inhibitory receptors (TIM‑3, TIGIT) and CD45RA, and between TIM‑3 and CD16 (Fig. 6a).

These correlations weakened or disappeared during anti-TNF therapy. Similarly, in the healthy control group, the associations were generally weaker (Fig. 6b). However, weak but specific correlations involving the DNAM-1 receptor were observed: a positive correlation with CD56 (p = 0.015, r = 0.370) and a negative correlation with CD45RA (p < 0.001, r = −0.557), which were not present in RA patients (Fig. 6b). These findings may reflect a state of physiological balance in γδ T cells in healthy individuals that is distinct from the dysregulated state observed in RA.

### Distinct correlations between receptors with divergent functions and clinical parameters in RA patients

Correlation matrices were generated to investigate the relationships between γδ T cells (overall), their Vδ1 and Vδ2 subsets, activating receptors, inhibitory receptors, and the clinical parameters of patients with RA. The analysis included both the proportion of γδ T cells and the median fluorescence intensity (MFI) values. The following clinical parameters were assessed: CRP, ESR, NTJ, NSJ, VAS, DAS, haemoglobin, creatinine, AST, and ALT.

The results revealed an intriguing pattern: γδ T cells overall, as well as the Vδ2 subpopulation, displayed negative correlations with clinical parameters, whereas the Vδ1 subpopulation exhibited positive correlations. Furthermore, there was a negative correlation between activating receptors (CD27, CD28 and DNAM-1), while there was a positive correlation between inhibitory receptors (TIGIT, TIM-3, PD-1) and clinical parameters.

It should be noted that our previous findings demonstrated reduced frequencies of γδ T cells and γδ T cells expressing activating receptors in RA patients, as well as a higher proportion of cells bearing inhibitory receptors. Considering this, the observed correlations are consistent with previous observations. However, as these correlations involve different clinical parameters at various time points during treatment, confirmation in a larger study cohort is warranted (see Supplementary Data Fig. S2-S7).

### γδ T cells and their receptors as a marker of effective therapy in RA

Based on previous analyses highlighting the significance of γδ T cells and their receptors in the response to anti-TNF therapy, the potential modulation of cell activity through receptor correlations, as well as associations between receptors and clinical parameters in RA patients, we performed ROC analyses.

This study aimed to evaluate the association of γδ T cells and their surface receptors with a favorable response to anti-TNF treatment. The analysis focused on patients who exhibited a successful response to treatment after six months, defined as a decrease in DAS28 score ≥ 1.2 and low disease activity at the endpoint.

Calculations were carried out for both the percentage of γδ T cells expressing a given receptor and the median fluorescence intensity (MFI). Analysis of parameters such as the area under the curve (AUC), statistical significance (set at p ≤ 0.05), and cut-off values determined based on sensitivity and specificity indicates the value of these data.

**Fig. 7:**
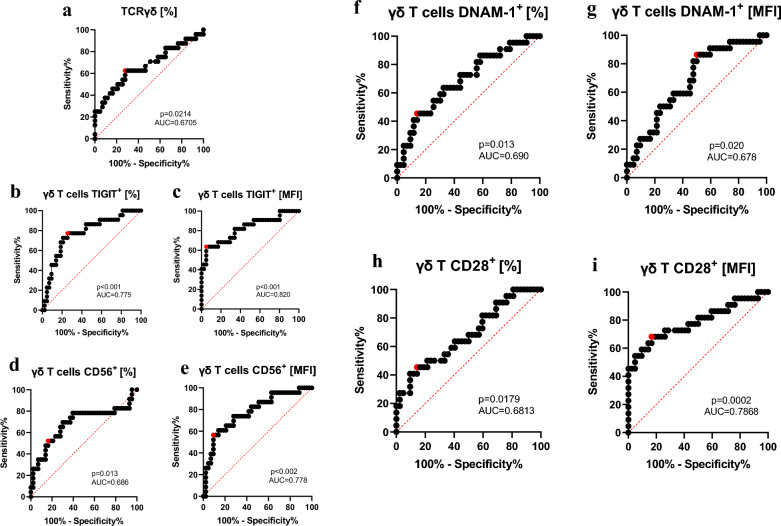
Receiver Operating Characteristic (ROC) curve for assessing the discriminatory potential of proteins in patients responding well to anti-TNF treatment compared to healthy controls. **a**,**b**, **d**, **f**, **h**) analysis of the percentage of cells expressing a given receptor; **c**, **e**, **g**, **i**) analysis of the MFI values for the respective receptor

Significant results were obtained based on the percentage of γδ T cells, with sensitivity and specificity of 62.5% and 72.09%, respectively (Fig. 7a). The most notable findings for both percentage and MFI were observed for CD56⁺ γδ T cells (Fig. 7d,e), DNAM-1⁺ γδ T cells (Fig. 7f,g), CD28⁺ γδ T cells (Fig. 7h,i), and TIGIT⁺ γδ T cells (Fig. 7b,c). The latter showed the highest sensitivity and specificity: 77.27% and 74.42% for cell percentage, and 63.64% and 95.12% for MFI, respectively.

The obtained results indicate that γδ T-cell frequency and surface receptor expression may reflect immunological features associated with a good response to anti-TNF therapy and could support treatment stratification in responding patients.

## Discussion

The Surface receptors of γδ T cells are a crucial factor in regulating their activity, as they can be activated independently of MHC. Therefore, understanding the role and functionality of γδ T cell receptors under both homeostatic conditions and pathological conditions could prove to be highly informative. This knowledge not only helps to elucidate the underlying mechanisms of various diseases but may also support the identification of novel diagnostic markers and the selection of more effective therapeutic strategies.

In this context, we analyzed the receptor profile of γδ T cells in patients with RA undergoing anti-TNF biological therapy, comparing our data with results obtained in healthy controls. Several key findings emerged from our study.

Firstly, we observed a reduced percentage of γδ T cells in the peripheral blood of RA patients compared to healthy controls. This finding corroborates previous reports showing similar trends in RA and in juvenile idiopathic arthritis (JIA) [22, 23]. The underlying biological rationale for this phenomenon is that the Vδ2 γδ T cell subpopulation has the capacity to migrate from the bloodstream into inflamed sites, such as joints [12]. In our study, we also noted a tendency—although not statistically significant—toward a decrease in circulating Vδ2 cells accompanied by a concomitant increase in Vδ1 cells, compared with the controls. This observation is consistent with the aforementioned mechanism. Lack of differences or reduced levels of Vδ2 cells in peripheral blood has also been reported elsewhere [12, 24, 25].

However, not all studies are consistent. Hassan et al. reported no significant differences in γδ T cell counts between RA patients and controls, but found an association with patient age [26]. We did not confirm such an age-dependent relationship in either RA patients or controls. However, other studies clearly suggest that the frequency of γδ T cells declines with age [27, 28].

Research on the impact of biological treatment on γδ T cells remains scarce. It has been demonstrated that high TNF concentrations contribute to Vδ2 chemotaxis and migration to inflamed sites, resulting in reduced peripheral counts [12]. A particularly intriguing case report described a patient with cutaneous γδ T-cell lymphoma (CGD-TCL) and underlying RA, in whom anti-TNF treatment with etanercept was associated with CGD-TCL development. Remarkably, discontinuation of etanercept led to complete regression of skin lesions, without relapse—an atypical course for this malignancy, strongly suggesting a causal link with therapy [29]. Other studies have indicated clonal expansion of γδ T cells following infliximab treatment, both in vitro and in patients with Crohn’s disease, which is another autoimmune condition [30]. Against this background, our observation of reduced γδ T cell percentages and MFI values may be associated with poorer or absent response to anti-TNF therapy. However, this finding requires validation in larger cohorts, given the generally favorable treatment response observed in our study population. Similarly, the negative correlation observed between γδ T cells (and Vδ2 cells specifically) and clinical parameters suggests that clinical improvement should parallel an increase in circulating γδ T cell frequencies.

The effector phenotype of γδ T cells can be defined by the expression of CD45RA and CD27. Telomere length analyses have revealed a differentiation trajectory from naïve CD45RA⁺CD27⁺ cells to central memory (TCM) CD45RA⁻CD27⁺ cells, followed by a transition to effector memory (TEM) CD45RA⁻CD27⁻ cells, and ultimately, terminally differentiated TEMRA CD45RA⁺CD27⁻ cells [31, 32]. We demonstrated that RA patients have fewer central memory cells but an increased proportion of terminally differentiated cells, which have limited proliferative potential but greater cytotoxic activity. The hypothesis was further supported by elevated frequencies and MFI values of CD56, a marker of cytotoxicity [33, 34]. A similar shift toward more differentiated Vδ2 cells has been reported by Guggino et al. [35]. TEMRA cells, often detected at inflammatory sites, produce perforins and granulysins, reinforcing their role in RA pathogenesis [33]. TEMRA cells with high CD45RA expression are also considered markers of T cell exhaustion [36].

Exhaustion typically develops in three stages, well-characterized in cancer and chronic viral infections, and we also consider it to be relevant to RA [37, 38]. Firstly, prolonged antigenic stimulation in RA primarily citrullinated proteins, inducing ACPAs and immune activation. Secondly, negative regulation through inhibitory receptor overexpression, consistent with our findings of increased frequencies and MFI values for TIGIT, TIM-3, and PD-1. Thirdly, the presence of chronic inflammation, a hallmark of RA, is particularly prevalent in patients qualifying for biological treatment due to high disease activity. Among inhibitory markers, PD-1 has been described most frequently. Compared to controls, Elevated PD-1 and PD-L1 expression has been observed both systemically and in synovial tissue in RA patients [39, 40]. The PD-1/PD-L1 axis is therefore central to RA pathogenesis, as also confirmed in CIA mouse models [40, 41]. In our study, TIGIT emerged as the most robust marker with the highest ROC values. However, it remains underexplored in γδ T cells in RA. Long et al. recently showed that in RA, CD8⁺ T cells with a TIGIT⁺KLRG1⁺ phenotype, which is associated with exhaustion and EOMES signature, are more frequent in patients lacking HLA-DR4 alleles, while being reduced in DR4 carriers. Importantly, abatacept (CTLA4-Ig) treatment selectively increased these cells in RA patients with risk alleles, suggesting a role for TIGIT⁺KLRG1⁺ TEX cells in differential immunotherapy responses [42].

Co-expression of TIGIT, TIM-3, and PD-1 on γδ T cells has so far been mainly described in cancer, where it signals exhaustion and immunotherapy potential [43–46]. It may be clinically relevant to identify exhausted TEMRA γδ T cells as excluding the CD27⁻CD45RA^hi subsets (exhausted cells) in favor of less differentiated populations of at least 1.5% could optimize preparations for adoptive γδ T cell immunotherapy [36].

Correlation analyses between γδ T cell receptor expression and clinical parameters revealed a general trend: negative associations for activating receptors, and positive ones for inhibitory receptors. Despite the absence of direct evidence in γδ T cells, similar findings have been reported in conventional T cells. For instance, TIGIT expression correlated positively with disease activity in RA patients [47]. However, this correlation has not been consistently replicated in all studies [48]. Increased serum TIM-3 [49, 50] and elevated PD-1 expression on conventional T cells in high DAS28 patients have also been documented [51]. Furthermore, a reduction in less-differentiated CD45RA⁻CD27⁺ γδ T cells, which has also been observed in RA, was found to be associated with higher disease activity in SLE [52]. Collectively, these correlations suggest a consistent pattern in which inhibitory and activating signals jointly modulate γδ T cell effector functions in RA.

Current RA diagnostic schemes primarily rely on ACR/EULAR criteria, combining clinical assessment, laboratory markers (RF, anti-CCP), and inflammation indices (ESR, CRP) [53]. Although these markers are clinically useful, they lack optimal sensitivity and specificity, meaning that some patients remain seronegative despite having active disease. Moreover, dynamic biomarkers are becoming increasingly valued for disease monitoring and treatment prediction. Although ACPA titers and CRP/ESR changes correlate with therapeutic response, their predictive precision remains limited [54]. In this context, novel immunological markers such as inhibitory receptor expression (PD-1, TIGIT, TIM-3) may serve as indicators of immune exhaustion and potential predictors of biological treatment outcomes [42, 55]. Our ROC analyses further emphasise the discriminatory potential of surface receptors, including TCRγδ itself, as possible predictors of anti-TNF response. This is a promising avenue for future research, although larger patient cohorts are necessary before such assays can be developed into clinically applicable diagnostic tools.

In summary, the present study provides the first comprehensive characterization of γδ T cells and their surface receptors in RA patients undergoing anti-TNF therapy. We demonstrated that (1) percentages of γδ T cells are reduced in RA, (2) γδ T cells acquire a terminally differentiated phenotype, consistent with exhaustion, (3) there is increased expression of inhibitory receptor, which further supports exhaustion, (4) receptors may serve as potential diagnostic and prognostic markers, (5) changes in percentage and MFI of γδ TCR may inform anti-TNF response assessment, and (6) receptor parameters correlate with clinical indices of disease activity.

These findings should, however, be interpreted in light of several limitations. Although no significant correlation between age and the analyzed immunological parameters was observed in our cohort, differences in age between RA patients and healthy controls may still represent a potential confounding factor, particularly given that such associations have been reported in other studies. Furthermore, the broad panel of analyzed surface receptors and clinical variables increases the complexity of the dataset and may limit straightforward interpretation of correlation analyses, raising the possibility of overinterpretation of some observed associations. Another limitation is the population specificity of the study, as it was conducted in a relatively homogeneous Polish cohort, which may restrict the generalizability of the findings.

Despite these limitations, this study represents one of the few comprehensive analyses integrating γδ T cell immunophenotyping with clinical parameters and response to anti-TNF therapy. The use of an extensive panel of markers enabled a multidimensional assessment of alterations within this cell population, and the obtained results provide a valuable foundation for further functional and validation studies, which may contribute to a better understanding of the role of γδ T cells in RA pathogenesis and their potential application as diagnostic and prognostic biomarkers.

## Supplementary Information

Below is the link to the electronic supplementary material.

Supplementary file1 (DOCX 696 KB)

## Data Availability

All data presented in this study are available from the lead contact upon reasonable request. Flow cytometry row data have been deposited at Zenodo.org as FCS files and are publicly available as of the date of publication (10.5281/zenodo.17405936).
