# Supplementary material for: Immune imbalance in rheumatoid arthritis: insights from γδ T cell receptor phenotyping
Source: Hum Cell. 2026 Jun 4;39(6):85. doi: 10.1007/s13577-026-01393-1 (PMC13236840; doi:10.1007/s13577-026-01393-1)
Supplement: Supplementary file 1 — Supplementary file1 (DOCX 696 KB) [file 13577_2026_1393_MOESM1_ESM.docx]

**Human Cell**

**Immune imbalance in rheumatoid arthritis: insights from γδ T cell receptor phenotyping**

**Sylwia Biały^1*^, Joanna Wielińska^1,2^, Kinga Maria Tyczyńska^3^,
Jerzy Świerkot^3^, Katarzyna Bogunia-Kubik^1^**

^1^Laboratory of Clinical Immunogenetics and Pharmacogenetics, Hirszfeld Institute of Immunology and Experimental Therapy, Polish Academy of Sciences, Wroclaw, Poland

^2^Department of General Biochemistry, University of Lodz, Lodz, Poland

^3^Department and Clinic of Rheumatology and Internal Diseases, Wroclaw Medical University, Wroclaw, Poland

*Correspondence: [sylwia.bialy@hirszfeld.pl](mailto:sylwia.bialy@hirszfeld.pl)

**Supplementary Data:**

| **a**   | **b**   |
| --- | --- |

Figure S1 No changes in the frequency of gamma delta T cells with the following phenotypes: a) naïve b) effector memory (TEM) were observed in RA patients during treatment or compared with the control group.

| **a**   |   **b** |
| --- | --- |
|   **c** | **d**   |
|   **e** |  |

Figure S2 Changes in A,B,C,E) the percentage and D) the MFI of A,B) activatory receptors, C,D) inhibitory receptors and E) cytotoxicity marker during anti-TNF treatment compared to healthy controls.

Table S1 Correlation matrix between the percentage of γδ T cells expressing the selected surface receptor and clinical parameters of RA patients before initiation of anti-TNF therapy. Statistically significant values are highlighted with bold frames in the table, and the corresponding p-values for these correlations are presented in the table below.
DAS28 – Disease Activity Score in 28 joints; NTJ – Number of Tender Joints; NSJ – Number of Swollen Joints; VAS – Visual Analogue Scale; Hb – Hemoglobin; ESR – Erythrocyte Sedimentation Rate; CRP – C-Reactive Protein; AST – Aspartate Aminotransferase; ALT – Alanine Aminotransferase; ns – not statistically significant.


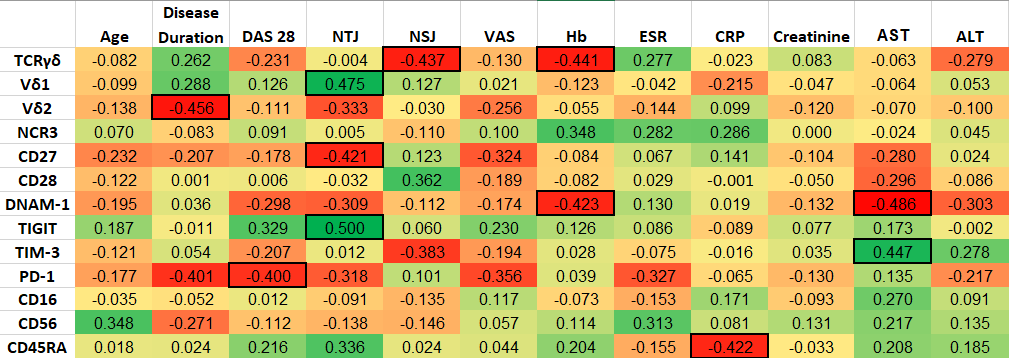

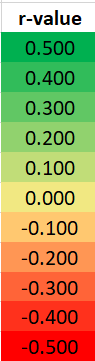


|  | Disease Duration | DAS 28 | NTJ | NSJ | Hb | CRP | AST |
| --- | --- | --- | --- | --- | --- | --- | --- |
| TCRγδ | ns | ns | ns | p=0.026 | p=0.018 | ns | ns |
| Vδ1 | ns | ns | p=0.012 | ns | ns | ns | ns |
| Vδ2 | p=0.017 | ns | ns | ns | ns | ns | ns |
| CD27 | ns | ns | p=0.036 | ns | ns | ns | ns |
| DNAM-1 | ns | ns | ns | ns | p=0.032 | ns | p=0.012 |
| TIGIT | ns | ns | p=0.013 | ns | ns | ns | ns |
| TIM-3 | ns | ns | ns | ns | ns | ns | p=0.022 |
| PD-1 | ns | p=0.043 | ns | ns | ns | ns | ns |
| CD45RA | ns | ns | ns | ns | ns | p=0.032 | ns |

Table S2 Correlation matrix between the MFI of γδ T cells expressing the selected surface receptor and clinical parameters of RA patients before initiation of anti-TNF therapy. Statistically significant values are highlighted with bold frames in the table, and the corresponding p-values for these correlations are presented in the table below.
DAS28 – Disease Activity Score in 28 joints; NTJ – Number of Tender Joints; NSJ – Number of Swollen Joints; VAS – Visual Analogue Scale; Hb – Hemoglobin; ESR – Erythrocyte Sedimentation Rate; CRP – C-Reactive Protein; AST – Aspartate Aminotransferase; ALT – Alanine Aminotransferase; ns – not statistically significant.


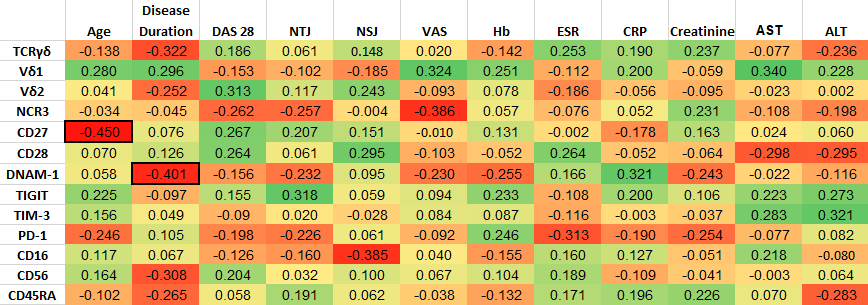

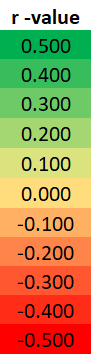


|  | Age | Disease Duration |
| --- | --- | --- |
| CD27 | p=0.019 | ns |
| DNAM-1 | ns | p=0.047 |

Table S3 Correlation matrix between the percentage of γδ T cells expressing the selected surface receptor and clinical parameters of RA patients after 3 months of anti-TNF therapy. Statistically significant values are highlighted with bold frames in the table, and the corresponding p-values for these correlations are presented in the table below.
DAS28 – Disease Activity Score in 28 joints; NTJ – Number of Tender Joints; NSJ – Number of Swollen Joints; VAS – Visual Analogue Scale; Hb – Hemoglobin; ESR – Erythrocyte Sedimentation Rate; CRP – C-Reactive Protein; AST – Aspartate Aminotransferase; ALT – Alanine Aminotransferase; ns – not statistically significant.


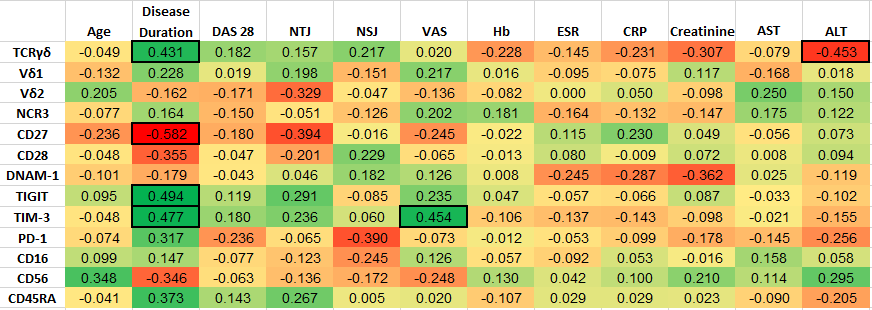

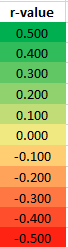


|  | Disease Duration | VAS | AST |
| --- | --- | --- | --- |
| TCRγδ | p=0.036 | ns | p=0.023 |
| CD27 | p=0.005 | ns | ns |
| TIGIT | p=0.020 | ns | ns |
| TIM-3 | p=0.025 | p=0.034 | ns |

*Table S4 Correlation matrix between the MFI of γδ T cells expressing the selected surface receptor and clinical parameters of RA patients after 3 months of anti-TNF therapy. Statistically significant values are highlighted with bold frames in the table, and the corresponding p-values for these correlations are presented in the table below.*DAS28 – Disease Activity Score in 28 joints; NTJ – Number of Tender Joints; NSJ – Number of Swollen Joints; VAS – Visual Analogue Scale; Hb – Hemoglobin; ESR – Erythrocyte Sedimentation Rate; CRP – C-Reactive Protein; AST – Aspartate Aminotransferase; ALT – Alanine Aminotransferase; ns – not statistically significant


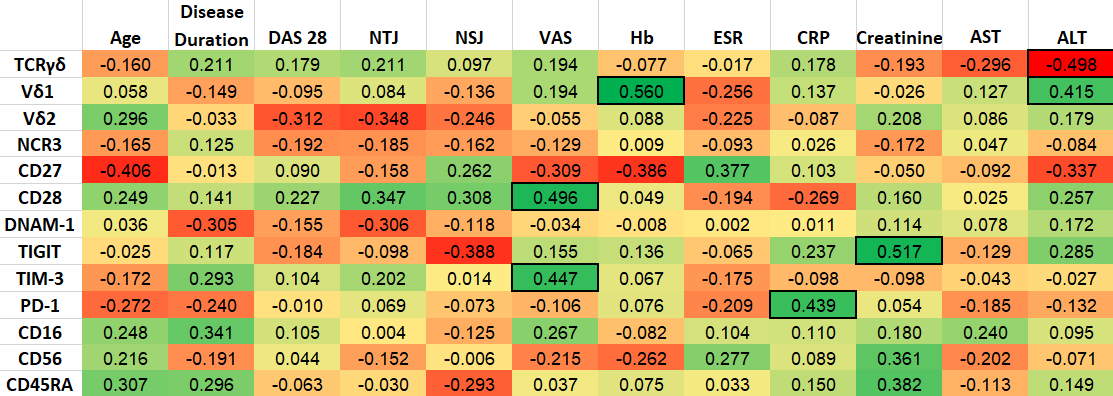

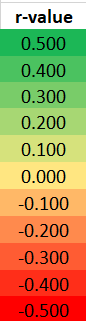


|  | VAS | Hb | CRP | Creatinine | ALT |
| --- | --- | --- | --- | --- | --- |
| TCRγδ | ns | ns | ns | ns | p=0.011 |
| Vδ1 | ns | p=0.007 | ns | ns | p=0.039 |
| CD28 | p=0.019 | ns | ns | ns | ns |
| TIGIT | ns | ns | ns | p=0.012 | ns |
| TIM-3 | p=0.037 | ns | ns | ns | ns |
| PD-1 | ns | ns | p=0.036 | ns | ns |

*Table S5 Correlation matrix between the percentage of γδ T cells expressing the selected surface receptor and clinical parameters of RA patients after 6 months of anti-TNF therapy. Statistically significant values are highlighted with bold frames in the table, and the corresponding p-values for these correlations are presented in the table below.*DAS28 – Disease Activity Score in 28 joints; NTJ – Number of Tender Joints; NSJ – Number of Swollen Joints; VAS – Visual Analogue Scale; Hb – Hemoglobin; ESR – Erythrocyte Sedimentation Rate; CRP – C-Reactive Protein; AST – Aspartate Aminotransferase; ALT – Alanine Aminotransferase; ns – not statistically significant


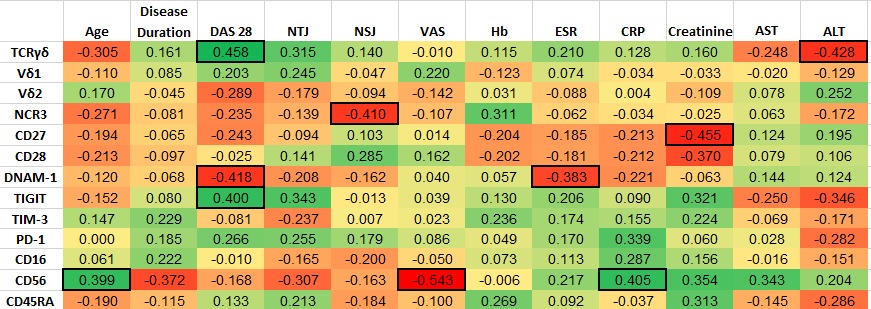

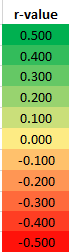


|  | Age | DAS 28 | NTJ | NSJ | VAS | ESR | CRP | Creatinine | ALT |
| --- | --- | --- | --- | --- | --- | --- | --- | --- | --- |
| TCRγδ | ns | p=0.013 | ns | ns | ns | ns | ns | ns | p=0.021 |
| NCR3 | ns | ns | ns | p=0.034 | ns | ns | ns | ns | ns |
| CD27 | ns | ns | ns | ns | ns | ns | ns | p=0.017 | ns |
| DNAM-1 | ns | p=0.030 | ns | ns | ns | p=0.049 | ns | ns | p=0.012 |
| TIGIT | ns | p=0.039 | ns | ns | ns | ns | ns | ns | ns |
| CD56 | p=0.039 | ns | ns | ns | p=0.003 | ns | p=0.036 | ns | ns |

*Table S6 Correlation matrix between the MFI of γδ T cells expressing the selected surface receptor and clinical parameters of RA patients after 6 months of anti-TNF therapy. Statistically significant values are highlighted with bold frames in the table, and the corresponding p-values for these correlations are presented in the table below.*DAS28 – Disease Activity Score in 28 joints; NTJ – Number of Tender Joints; NSJ – Number of Swollen Joints; VAS – Visual Analogue Scale; Hb – Hemoglobin; ESR – Erythrocyte Sedimentation Rate; CRP – C-Reactive Protein; AST – Aspartate Aminotransferase; ALT – Alanine Aminotransferase; ns – not statistically significant


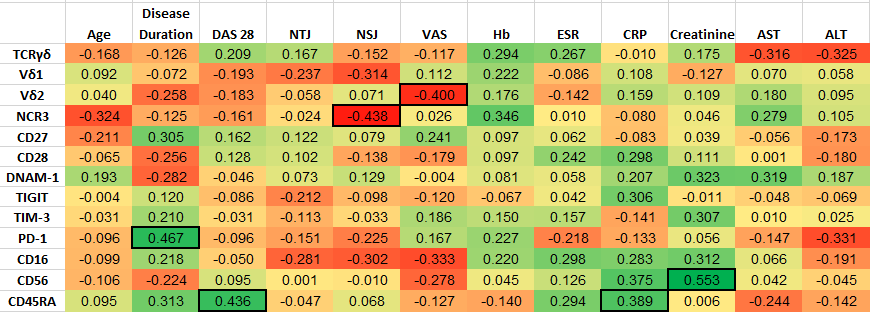

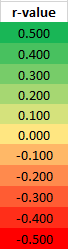


|  | Disease Duration | DAS 28 | NSJ | VAS | CRP | Creatinine |
| --- | --- | --- | --- | --- | --- | --- |
| Vδ2 | ns | ns | ns | p=0.032 | ns | ns |
| NCR3 | ns | ns | p=0.025 | ns | ns | ns |
| PD-1 | p=0.019 | ns | ns | ns | ns | ns |
| CD56 | ns | ns | ns | ns | ns | p=0.003 |
| CD45RA | ns | p=0.026 | ns | ns | p=0.050 | ns |
